# Improvement of a tissue maceration technique for the determination of placental involvement in schistosomiasis

**DOI:** 10.1371/journal.pntd.0005551

**Published:** 2017-04-24

**Authors:** Martha Charlotte Holtfreter, Heinrich Neubauer, Tanja Groten, Hosny El-Adawy, Jana Pastuschek, Joachim Richter, Dieter Häussinger, Mathias Wilhelm Pletz, Benjamin Thomas Schleenvoigt

**Affiliations:** 1 Tropical Medicine Unit, Department of Gastroenterology, Hepatology and Infectious Diseases, Faculty of Medicine, Heinrich-Heine-University, Düsseldorf, Germany; 2 Friedrich-Loeffler-Institut, Federal Research Institute for Animal Health, Institute of Bacterial Infections and Zoonoses, Jena, Germany; 3 Department of Gynaecology and Obstetrics, Jena University Hospital, Jena, Germany; 4 Institute of Tropical Medicine and International Public Health, Charité Universitätsmedizin, Berlin, Germany; 5 Center for Infectious Diseases and Infection Control, Jena University Hospital, Jena, Germany; George Washington University, UNITED STATES

## Abstract

Schistosomiasis in pregnancy may cause low birth weight, prematurity and stillbirth of the offspring. The placenta of pregnant women might be involved when schistosome ova are trapped in placental tissue. Standard histopathological methods only allow the examination of a limited amount of placental tissue and are therefore not sufficiently sensitive. Thus, placental schistosomiasis remains underdiagnosed and its role in contributing to schistosomiasis-associated pregnancy outcomes remains unclear. Here we investigated an advanced maceration method in order to recover a maximum number of schistosome ova from the placenta. We examined the effect of different potassium hydroxide (KOH) concentrations and different tissue fixatives with respect to maceration success and egg morphology. Placental tissue was kept either in 0.9% saline, 5% formalin or 70% ethanol and was macerated together with *Schistosoma mansoni* infested mouse livers and KOH 4% or 10%, respectively. We found that placenta maceration using 4% KOH at 37°C for 24 h was the most effective method: placental tissue was completely digested, egg morphology was well preserved and alkaline concentration was the lowest. Ethanol proved to be the best fixative for this method. Here we propose an improved maceration technique in terms of sensitivity, safety and required skills, which may enable its wider use also in endemic areas. This technique may contribute to clarifying the role of placental involvement in pregnant women with schistosomiasis.

## Author summary

Schistosomiasis in pregnant women is associated with prematurity, low birth weight and stillbirth of the fetus. Schistosome eggs may be trapped in the placental tissue and, thus, contribute to fetal harm. As the placenta is a large organ, current microscopic histopathological examinations commonly lack sensitivity. The yield of schistosome eggs can be increased by the use of tissue maceration. However, the applied maceration procedures are labor intensive, time-consuming and cumbersome. To develop an improved maceration technique in terms of sensitivity, safety and required skills which enable its wider use in endemic areas we examined the effect of different potassium hydroxide (KOH) concentrations and different tissue fixatives with respect to maceration success and egg morphology. Placenta maceration using 4% KOH at 37°C for 24 h was the most effective method: placental tissue was completely digested, egg morphology was well preserved and alkaline concentration was the lowest. This improved technique may contribute to clarify the role of placental involvement in pregnant women with schistosomiasis.

## Introduction

Schistosomiasis may involve the placenta of pregnant women when ova are trapped in placental tissue. This has been described in both *Schistosoma haematobium* and *Schistosoma mansoni* (*S*. *mansoni*) infections [1, 2, 3, 4, 5]. Lesions are predominantly located in the placental villi, the intervillous space and the decidua [6, 7]. Ova can be found with or without surrounding granulomatous inflammations [7].

Although placental involvement of schistosomiasis has been described in the literature since the beginning of the 20^th^ century, only few cases have been published so far [1, 3, 4, 5, 6]. Diagnostic proof of schistosome ova in placental tissue is normally performed by investigation of histological cross sections. As the placenta is a large organ of which only a limited volume can be routinely analyzed and the tissue density of schistosome ova is usually low, routine histopathological examinations are not sufficiently sensitive.

Maceration techniques aim to remove soft tissue by the destruction of biomolecules. Common methods include the use of inorganic chemicals like sodium hydroxide, ammonium hydroxide and other alkaline solutions [8]. In the early 1970s a tissue maceration technique using 10% potassium hydroxide (KOH) was described to screen larger placenta specimens for schistosome ova [2]. In the studies of Sutherland *et al*. (1965) and Renaud *et al*. (1972) a total number of 322 placentas were examined for schistosome ova. By use of the maceration method, ova were found in 79 cases whereas only two cases had been detected by histological examinations. However, the applied maceration procedures are labor intensive, time-consuming and cumbersome. Therefore, this technique was rarely used in recent studies or routine diagnosis.

The aim of this study was to develop an improved maceration technique that can easily be applied, allows the examination of larger naïve and fixed placenta specimens and improves the sensitivity for ova detection. Thus, we examined the effect of different tissue fixatives on the maceration success and the effect of different potassium hydroxide concentrations on the morphology of schistosome ova.

## Materials and methods

### Ethics statement

All women signed an informed consent allowing the use of the placental tissue for scientific purposes. The scientific use of these residual samples was approved by the Ethical Committee of the University Hospital Jena, Germany.

### Specimens

Placental tissues were obtained from healthy women who delivered spontaneously or by caesarean section.

Two livers from *Schistosoma mansoni* (Puerto Rican strain) infected Swiss-Webster female mice were kindly provided by the Biomedical Research Institute (BRI), Rockville, MD 20852. Livers were kept refrigerated in 0.9% saline.

### Effect of different tissue fixatives on the maceration process

The placenta was cut into several approximately 5 x 5 cm large specimens as described by Renaud *et al*. (1972) and kept either in 0.9% saline, 5% formalin or 70% ethanol. Each placenta specimen was cut into several smaller specimens (1 x 1 cm) that were placed in a 50 mL tube. Subsequently, 10% potassium hydroxide (KOH) was added until a final volume of 45 mL was achieved. Tubes were gently shaken to allow the 10% KOH to cover all of the placental tissue. The 50 mL tubes were placed in a warming cabinet and incubated for 24 h at 37°C. The caps were not tightened but placed onto the tube in order to allow developing gases to escape. After 24 h the test tubes were checked for maceration success. All experiments were performed in duplicates.

### Effect of different potassium hydroxide concentrations on the morphology of schistosome ova

As we observed an incomplete maceration process of the 5% formalin fixed placenta tissues after 24 h, specimens used for this experiment were conserved either in 0.9% saline or 70% ethanol. As described above one placenta piece was cut into several smaller pieces and placed in a 50 mL test tube. Two *Schistosoma mansoni* infected naïve mouse livers were cut into approximately 0.5 x 0.5 cm pieces and three pieces from different liver areas were added to each tube with placental tissue, respectively. Tubes were filled with either 10% KOH or 4% KOH and placed in a warming cabinet for 24 h at 37°C as described above. Mouse liver pieces incubated alone with 10% KOH or 4% KOH served as controls to verify that schistosomal eggs were not digested by KOH. After 24 h the test tubes were centrifuged for 10 minutes at 2,500 rpm and room temperature. The supernatants were discarded and the whole pellets were examined under the microscope at 100-fold and 400-fold magnification for eggs of *Schistosoma* sp. with particular attention to their morphology. The experiments were performed in duplicates.

### Limitations

No quantitative assessment of the eggs trapped in the mouse liver pieces was made. Therefore no experimental data about the lower limit of detection of schistosome ova in placenta pieces were collected.

## Results

### Effect of different tissue fixatives on the maceration process

After 24 hours the placental tissue conserved either in 0.9% saline or 70% ethanol was completely digested. Placental tissue fixed in 5% formalin was not fully macerated as the tube still contained a large number of undigested tissue pieces.

### Effect of different potassium hydroxide concentrations on the morphology of schistosome ova

**Fig 1:**
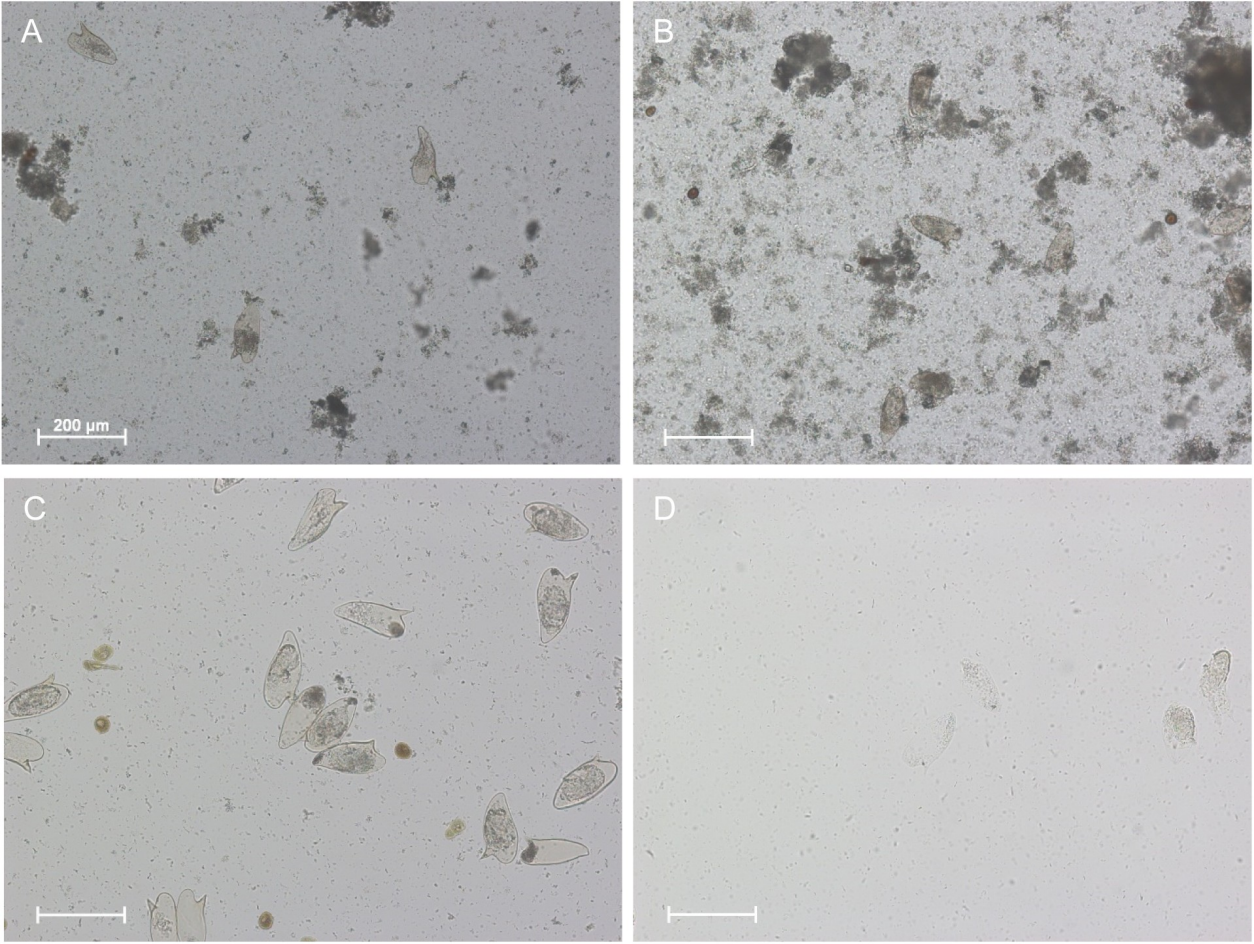
Preservation of egg morphology after incubation with different fixatives and KOH concentrations. Incubation of 70% ethanol-fixed placental tissue (A) or naive placental tissue (B) and infected mouse liver with 4% KOH for 24h at 37°C. Mouse liver pieces incubated alone with 4% KOH (C) or 10% KOH (D) served as controls. In all groups incubated with 4% KOH the eggshells of nearly all eggs of *S*. *mansoni* were intact and the lateral spine was easy to identify. The eggs contained a more or less developed miracidium or were without any content. *S*. *mansoni* eggs in the 10% KOH control group were nearly completely digested. The morphology and the lateral spine were hard to identify.

After 24 hours *S*. *mansoni* ova were found in all preparations. In all experimental groups with 0.9% saline + 10% KOH or 4% KOH, and 70% ethanol + 10% KOH or 4% KOH the shells of almost all ova were intact and the lateral spine was clearly visible (Fig 1A and 1B). Ova contained either a more or less decomposed miracidium or were without content. When mouse livers were directly incubated with 10% KOH all ova were nearly completely digested and detection of the lateral spine or the miracidium was challenging (Fig 1D). Egg morphology was preserved when mouse liver tissue was incubated with 4% KOH as observed in the experimental groups (Fig 1C).

## Discussion

In our experiments we found that placenta maceration using 4% KOH at 37°C for 24 h was the most effective method: placental tissue was completely digested, egg morphology was well preserved and alkaline concentration was lowest. In previous studies placental tissue was commonly digested in 10% KOH at 37°C [1, 4] or 56°C [2,3] for 24–48 hours. However, Renaud *et al*. (1972) reported, that it was usually impossible to determine the schistosomal species in digested placental tissue as the egg shape was often altered by the potassium hydroxide and the spine was not visible. In animal experiments digestion with 4% KOH for 24–48 hours was previously described for the extraction of trapped eggs in different tissues though this procedure was never used in human samples for diagnostic purpose [9].

Renaud *et al*. (1972) and Gelfand *et al*. (1970, 1971) used 250 mL glass receptacles or glass bottles, collected the sediment by a pipette and transferred it into centrifuge tubes. However, collection of the whole sediment is hard to ensure and the transfer of potassium hydroxide is hazardous for the personnel because of the risk of chemical burns. Furthermore, Renaud *et al*. (1972) described that the maceration process was incomplete when using 2 x 22 cm glass tubes. Therefore, in our experiments, we used disposable 50 mL tubes for the maceration and subsequent centrifugation. The tubes are easy to handle and preclude the loss of macerated tissue during the transfer process from receptacle bottom to centrifuge tube. By mincing the placental tissue the complete maceration was achieved as smaller pieces may offer a larger contact surface for the KOH solution.

Contrary, the complete digestion of 5% formalin fixed placenta samples failed in our experiments as tissue pieces were still found after 24 hours. This confirms a similar observation made in animal experiments (Tucker *et al*. 2013). Surprisingly, Gelfand *et al*. (1970, 1971) described the complete digestion of tissue samples that were fixed in 10% formalin. However, several washing steps were applied before application of KOH and a much higher incubation temperature of 56°C was used for the maceration process.

Schistosomiasis has been implicated in fetal harm including prematurity, low birth weight and stillbirth [6, 10, 11, 12, 13, 14]. The physiopathology of negative pregnancy outcomes in schistosomiasis may be multifactorial. Pregnant women with chronic schistosomiasis are known to be more frequently undernourished, anemic due to chronic iron loss, to suffer from chronic protein loss and vitamin deficiencies [15]. The role of placental involvement is not sufficiently elucidated as only a few studies have been undertaken. In two earlier studies from South Africa and the Ivory Coast a total of 343 placentas from both, normal deliveries and miscarriages, premature births or intrauterine fetal deaths were examined by digestion with 10% KOH [1, 4]. Schistosome ova were found in 79 cases but there was no evidence that placental schistomiasis had any impact on the pregnancy outcome or the development of the embryo or of the fetus. However, a symptomatic intestinal or urogenital schistosomiasis was not an inclusion criterion in this study and serology to detect an asymptomatic chronic infection was not performed, so that the percentage of placental involvement during a florid schistosomiasis could not be determined with sufficient accuracy. On the other hand there are several case reports describing that placental schistosomiasis was associated with various complications e.g. tubal pregnancy and stillbirth diagnosed by retrospective histological examinations of placental tissue [6].

Due to the described diagnostic difficulties, it has not been sufficiently investigated whether adverse birth events might be induced by a local inflammation process of the placenta caused by trapped eggs or with complex generalized inflammation due to the helminth infection resulting in increased inflammatory cytokines like tumor necrosis factor α (TNF- α) and interleukin 6 (IL-6) [13,16]. As studies have shown *in vitro*, first trimester trophoblasts (HTR8/SVneo cell line) showed significantly higher levels of the pro-inflammatory cytokines IL-6 and IL-8 after incubation with serum obtained from *S*. *japonicum* infected women at 32 week gestation in comparison to an uninfected control group [17]. Furthermore, incubation of the HRT8/SVneo cell line and placental cytotrophoblasts with soluble egg antigen (SEA), that was found circulating during schistosome infection, inhibited the migratory and invasive properties of the extravillous trophoblasts and also increased the pro-inflammatory cytokine levels [17, 18]. These data indicate that schistosomal antigen can activate pro-inflammatory response and might influence fetal health.

Contrary, in two recent studies from the Philippines and Uganda the effects of praziquantel treatment on the perinatal outcome of *S*. *japonicum* or *S*. *mansoni* infected mothers were examined [19, 20]. Both studies revealed that the treatment with praziquantel in the second or third trimester of gestation did not have a significant effect on the birthweight of the newborns nor on the haemoglobin levels of the mothers. However, in none of the studies the placentas were examined regarding schistosome ova and therefore no prediction can be made about the effect of placental schistosomiasis on the pregnancy outcome.

In summary, placental schistosomiasis remains underdiagnosed if examined by standard histopathological methods which are not sufficiently sensitive. Future investigations on schistosomiasis in pregnancy should include placenta maceration in order to establish the role of placental involvement especially in schistosomiasis-endemic settings. We have developed an improved maceration technique in terms of sensitivity, safety and required skills which enable its wider use in endemic areas.
